# Examination of Oral Functions Related to Excessive Daytime Sleepiness

**DOI:** 10.7759/cureus.98283

**Published:** 2025-12-02

**Authors:** Atsumi Sunakawa, Yoshiaki Ihara, Hirotaka Kato, Akira Minoura, Kojiro Hirano, Kouzou Murakami, Yoshio Watanabe, Yoshinori Ito, Akatsuki Kokaze

**Affiliations:** 1 Department of Oral Functional Rehabilitation Medicine, Showa Medical University Graduate School of Dentistry, Tokyo, JPN; 2 Department of Oral Health Management, Division of Oral Functional Rehabilitation Medicine, Showa Medical University, Tokyo, JPN; 3 Department of Hygiene, Public Health and Preventive Medicine, Showa Medical University School of Medicine, Tokyo, JPN; 4 Department of Otorhinolaryngology Head and Neck Surgery, Showa Medical University School of Medicine, Tokyo, JPN; 5 Department of Radiology, Division of Radiology, Showa Medical University School of Medicine, Tokyo, JPN; 6 Department of Medicine, Division of Respirology and Allergology, Showa Medical University School of Medicine, Tokyo, JPN; 7 Department of Radiology, Division of Radiation Oncology, Showa Medical University School of Medicine, Tokyo, JPN

**Keywords:** eating assessment tool-10, excessive daytime sleepiness, lip-seal strength, oral function, tongue pressure

## Abstract

Excessive daytime sleepiness (EDS) is a major occupational health concern that negatively affects worker safety, productivity, and overall health. Possible associations between oral function and EDS have been reported. This study aimed to investigate the relationships between lip-seal force, tongue pressure, and questionnaire-based assessments of oral function and EDS in a working-age population. A cross-sectional study was conducted with 392 Japanese male workers. Daytime sleepiness was assessed using the Epworth Sleepiness Scale (ESS), and swallowing function was evaluated using the Eating Assessment Tool-10 (EAT-10). Participants were classified into two categories based on ESS scores: a severe EDS group (ESS ≥11) and a moderate-to-severe EDS group (ESS ≥5). Additional variables included body mass index (BMI), self-reported snoring, tongue pressure, and lip-seal strength. To identify factors independently associated with higher ESS scores, we performed multivariate logistic regression analyses. Participants with ESS scores ≥11 were classified as having severe EDS, while those with scores ≥5 were categorized as having moderate or severe EDS. In the severe EDS group, EAT-10 scores and snoring were significantly associated with ESS. In the moderate or severe EDS group, significant associations were found between ESS scores and EAT-10, snoring, and lip-seal strength. No significant associations were observed between ESS scores and tongue pressure or BMI. The findings show that dysphagia and reduced lip-seal strength are significantly associated with EDS among working-age Japanese men. Oral function assessments may be a useful tool for early detection and prevention of sleep-related disorders in occupational health settings.

## Introduction

Excessive daytime sleepiness (EDS) is a major occupational health issue, impacting work performance and increasing accident risk [1]. EDS not only reflects symptoms of insomnia but also negatively affects workers' job productivity. Moreover, it is a major occupational health issue that increases the risk of traffic accidents, and measures are needed to address it.

Obstructive sleep apnea (OSA), which is often underdiagnosed because of its subtle symptoms, contributes substantially to EDS. Age-related decline in tongue muscle function increases the risk of OSA [2]. Early detection of OSA is crucial for the prevention of systemic conditions, such as hypertension [3].

Extended working hours, especially in aging labor forces, further increase EDS risk [4,5]. Maintaining a healthy work environment may reduce EDS and enhance productivity. Recent research has identified links between oral functions, such as lip-seal strength, and sleepiness [6]. Lip-seal strength reflects mastication and swallowing capacity, while both very short and long sleep durations are associated with poor oral health [7].

Pediatric oral malformations have also been associated with adult sleep disorders [8]. Thus, evaluating oral function, including lip closure and tongue pressure, may offer a non-invasive method to assess EDS risk. We previously reported that lip-seal strength and tongue pressure decrease with age and are influenced by eating habits in Japanese workers [9]. Based on previous findings, we hypothesized that specific oral functions, particularly lip-seal strength and tongue pressure, may be associated with EDS in working-age men.

## Materials and methods

Study design and population

This cross-sectional study was conducted between November 2021 and August 2022. Participants were recruited from various workplaces in Japan. The inclusion criteria were healthy adult males aged <65 years who were able to work. In total, 412 individuals consented to participate in this study. This study excluded participants with missing data (e.g., those who did not record their weight, had incomplete questionnaire responses, etc.). After excluding 20 participants owing to missing data or measurement outliers, the final sample consisted of 392 individuals with a mean age of 41.6 years (range: 19-64 years).

The exclusion criteria included a medical history of conditions affecting eating or swallowing (e.g., cerebrovascular disease and neuromuscular disorders), oral symptoms on the day of measurement (e.g., ulcers and tooth pain), and a prior diagnosis of sleep apnea. Prior to enrollment, all participants were informed about this study orally and in writing, and written informed consent was obtained.

This study was approved by the Showa University Ethics Review Committee of the Showa University School of Medicine (approval No. 21-088-A, October 8, 2021) and was conducted in accordance with the World Medical Association Declaration of Helsinki (version 2002).

Measurement comments

A self-administered questionnaire was used to collect data regarding daytime sleepiness, subjective swallowing function, weight (kg), height (cm), and snoring habits (yes/no). Tongue pressure and lip-seal strength were measured using specific instruments. All physical measurements were performed by the same dentist. A dentist who was certified by the Japanese Society of Swallowing Rehabilitation performed the measurements.

Daytime Sleepiness

Daytime sleepiness was assessed using the Epworth Sleepiness Scale (ESS). The ESS is a questionnaire used to assess subjective daytime sleepiness and its severity. It evaluates the presence of sleepiness in eight daily life situations, such as reading, on a four-point scale (0: none at all to 3: very high), with a maximum total score of 24. Higher total scores indicate stronger daytime sleepiness. A score of 11 points or higher indicated EDS [10,11]. We cited and used the Japanese version of the Epworth Sleepiness Scale reported by Fukuhara. This report is open access [12].

More severe OSA manifests as stronger EDS [13]; hence, we defined participants with an ESS score of 11 or higher as the severe EDS group and those with an ESS score of 5 or higher as the moderate and severe EDS group.

Subjective Swallowing Function Assessment

The Eating Assessment Tool-10 (EAT-10) was used to examine subjective swallowing function [14]. The EAT-10 is a screening tool for feeding and swallowing disorders that is rated on a five-point scale ranging from 0 to 4 for each of the 10 questions. The maximum score is 40 points, and a total score of 3 or more points is considered suspicious for dysphagia. We obtained formal permission from the Mapi Research Trust to use the EAT-10 and utilized the Japanese version of the EAT-10 that was distributed.

Body Mass Index

Body mass index (BMI) was calculated as weight (kg) divided by height squared (m²).

Perceived or Noted Snoring

Participants were asked whether they were aware of snoring during sleep or if they had been told by others that they had snored. Responses were recorded as either “yes” or “no.”

Tongue Pressure

**Figure 1:**
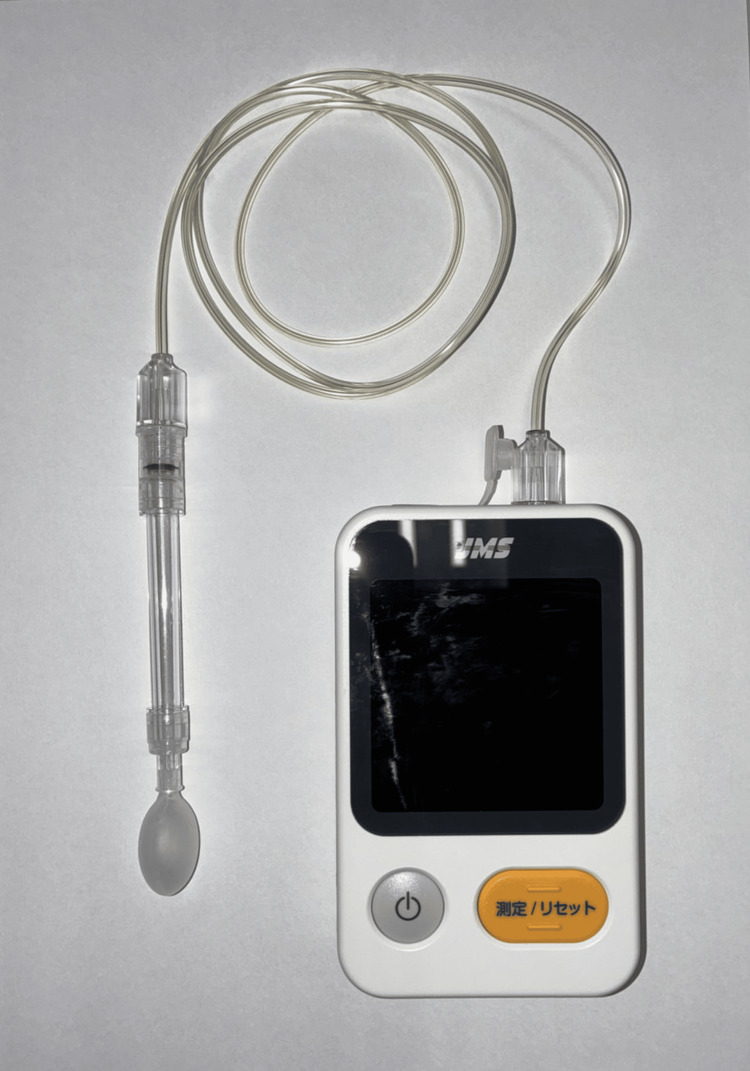
The TPM-01 device used for measuring tongue pressure

We used the TPM-01 device to measure tongue pressure (JMS Co. Ltd., Hiroshima, Japan) (Figure 1).

This instrument measures maximum pressure by pressing a probe-tip balloon, which is fed with air pressure, against the palate with the force of the tongue. Before the measurement, the participants were seated on a chair with the Frankfurt plane parallel to the floor. The participants were instructed to press the balloon against the hard palate using only the muscular force of the tongue and not to move the head or aspirate the balloon during the measurement.

The balloon tip of the tongue pressure probe was positioned at the center of the tongue. The participants were instructed to close their lips and press the balloon on the tongue against the hard palate with maximum force for seven seconds, and the maximum tongue pressure indicated by the tongue pressure measuring device was measured. We measured the participants’ maximum tongue pressure (kPa) three times and used the average.

Lip-Seal Strength

**Figure 2:**
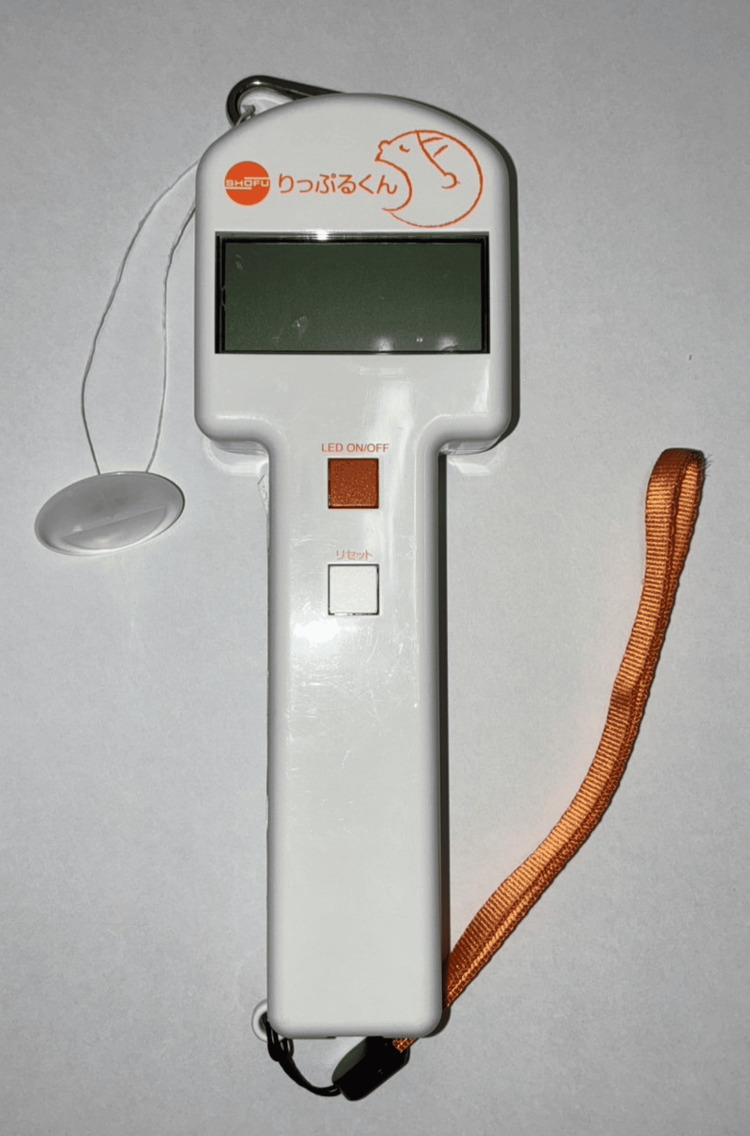
The device used for measuring lip-seal strength

In this study, we used a lip-seal strength measuring device called “Ripple-kun” (Shofu, Kyoto, Japan) (Figure 2).

This device has been reliable in past studies [15]. It is also used as a training device to improve lip-closure function [16]. Before measurement, the participants were instructed to resist the traction force only with lip-seal strength, not with the teeth, tongue, or suction, and not to move their heads during measurement. The button used for the measurement was positioned in the incisor-oral vestibule, and the lips were closed to ensure that the center of the button was positioned on the midline of the maxillary anterior teeth. The button and Ripple-kun were connected to a 30 cm dental floss. We pulled the Ripple-kun horizontally, parallel to the floor, and the maximum value was recorded until the button was removed from the participants’ lips. The participants’ lip-seal strength (N) was measured three times, and the average was used.

Participants completed the ESS and EAT-10 questionnaires before undergoing tongue pressure and lip-seal strength measurements. All physical measurements were performed by the same dentist.

Statistical analysis

For the obtained variables, the Shapiro-Wilk test was first performed to check whether they were normally distributed, and if they were considered normally distributed, the mean and standard deviation (SD) were obtained.

Otherwise, median (Mdn), first quartile (1Q), and third quartile (3Q) values were calculated. In addition, multivariate logistic regression analysis was performed to determine the effect of other variables on the ESS, the objective variable of this study, to obtain odds ratios and 95% confidence intervals. Statistical significance was set at p<0.05. The JMP 16.2 (SAS Institute, Inc., Cary, North Carolina) statistical analysis software was used.

## Results

After excluding 20 participants owing to missing data or outliers, 392 participants were included in the final analysis. The participant characteristics are shown in Table 1. 

**Table 1:** Characteristics of study participants Values for continuous variables are presented as medians with 25th and 75th percentiles unless otherwise indicated. Tongue pressure is presented as the average value. Snoring (yes) indicates the number of participants who reported snoring. BMI, body mass index; ESS, excessive daytime sleepiness; EAT-10, Eating Assessment Tool-10.

Characteristics	Mean (± standard deviation)	Max	Min
Age	44.4 (28.6, 52.7)	64	19
BMI (kg/m^2^)	23.7 (21.3, 26.7)	44.1	15.3
ESS (score)	5 (2.8)	21	0
Snoring (yes)	165		
EAT-10 (score)	(0, 0)	8	0
Tongue pressure (kPa)	41.5 (8.6)	61	15.6
Lip-seal strength (N)	13.4 (11.6, 15.7)	29.9	6.6

**Figure 3:**
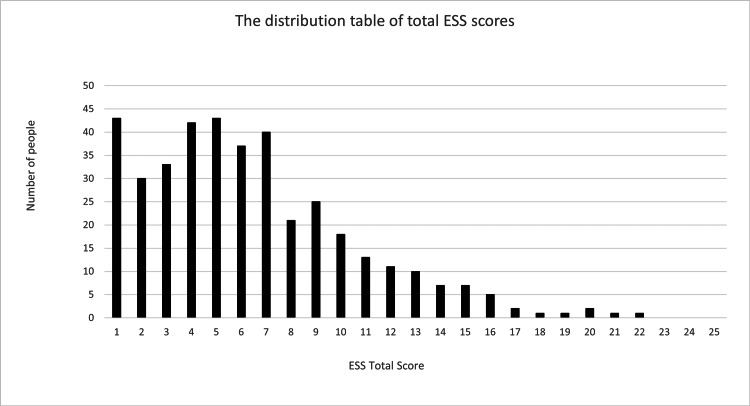
Distribution table of total Epworth Sleepiness Scale scores The horizontal axis is the total score, and the vertical axis is the number of people. ESS, Epworth Sleepiness Scale.

Among the measured variables, only tongue pressure was normally distributed, whereas all other variables showed non-normal distributions. Snoring was reported as a binary variable and presented as frequency (number of participants). Therefore, tongue pressure is presented as the mean (SD), and other variables are expressed as the median (25th-75th percentile). Figure 3 shows the distribution of the total ESS scores based on the self-administered questionnaire.

The most frequently reported scores were 0 and 4, with each score being recorded for 42 participants. No participants recorded an ESS score of 22 or higher. A total of 48 participants had an ESS score of 11 or higher, and 201 participants had a score of 5 or higher.

Based on these results, in this study, 48 participants with an ESS score ≥11 were classified as the severe EDS group, and 201 participants with a score ≥5 were classified as the moderate or severe EDS group.

Table 2 shows the results of the multivariate logistic regression analysis of the relationship between EDS and other variables in the severe EDS group. This table shows odds ratios, 95% confidence intervals, and significant differences. Significant associations were observed between the EAT-10 scores (p<0.0001) and snoring (p<0.001). In contrast, no significant associations were found for BMI, tongue pressure, or lip-seal strength, with p-values of 0.50, 0.25, and 0.34, respectively.

**Table 2:** Logistic regression analysis results for the severe excessive daytime sleepiness group and other variables *p < 0.05. OR, odds ratio; BMI, body mass index; EAT-10, Eating Assessment Tool-10.

Variables	OR	95% Confidence interval		p value
		Lower	Upper	
BMI (kg/m^2^)	0.97	0.89	1.06	0.5
Snoring (yes)	3.3	1.54	7.1	0.001*
EAT-10 (score)	1.59	1.27	1.98	<0.0001*
Tongue pressure (kPa)	0.98	0.94	1.02	0.25
Lip-seal strength (N)	0.95	0.85	1.06	0.34

Table 3 shows the results of the multivariate logistic regression analysis of the relationship between EDS and other variables in the moderate and severe EDS groups.

**Table 3:** Logistic regression analysis results for the moderate or severe excessive daytime sleepiness group and other variables *p < 0.05. OR, odds ratio; BMI, body mass index; EAT-10, Eating Assessment Tool-10.

Variables	OR	95% Confidence interval		p value
		Lower	Upper	
BMI (kg/m^2^)	0.99	0.93	1.05	0.74
Snoring (yes)	2.5	1.6	3.88	< .0001*
EAT-10 (score)	1.37	1.07	1.74	0.0052*
Tongue pressure (kPa)	1	0.98	1.04	0.51
Lip-seal strength (N)	0.85	0.79	0.92	< .0001*

Similar to Table 2, Table 3 shows the odds ratios, 95% confidence intervals, and significant differences. The moderate or severe EDS group had higher EAT-10 scores, and a higher proportion of participants snored compared to the group with ESS scores less than 5. Significant differences were found in EAT-10 scores, snoring, and lip-seal strength (p=0.0052, p<0.001, and p<0.001, respectively). In contrast, no significant differences were found between BMI and tongue pressure (p=0.74 and p=0.51, respectively).

## Discussion

This cross-sectional study aimed to explore the association between oral function, including lip-seal strength and tongue pressure, and EDS in healthy Japanese working-age men. The main findings indicated that both subjective swallowing difficulty, as measured using the EAT-10, and the presence of snoring were strongly associated with both moderate and severe EDS. Additionally, reduced lip-seal strength was significantly associated with moderate or severe EDS, whereas tongue pressure and BMI did not show statistically significant associations.

Phenbuny et al. reported that after training the tongue and orbicularis oris muscles for two months in OSA patients, ESS scores decreased. However, there are few studies that comprehensively investigate EDS and oral function, leading to discrepancies in reports across different papers [17].

These findings provide novel insights into the multifaceted relationship between oral physiology and sleep quality. EDS is a known risk factor for traffic accidents [18], occupational injury, and reduced work performance [19], and early identification of EDS is crucial in occupational settings. This study demonstrates that easily measurable oral function indicators, such as lip-seal strength and self-reported swallowing difficulty, may serve as useful markers for identifying individuals at a higher risk of experiencing daytime sleepiness, possibly due to undiagnosed sleep-related breathing disorders, including OSA [6,20].

Swallowing dysfunction and EDS

A robust and consistent association was observed between elevated EAT-10 scores and EDS in both severity groups. This suggests that even mild subjective symptoms of dysphagia may be associated with underlying oropharyngeal muscle dysfunction, which can compromise upper airway stability during sleep [21]. Impaired swallowing function may share neuromuscular and structural pathophysiology with OSA, including weakened pharyngeal muscles and altered tongue posture. Patients with OSA often display subtle signs of swallowing impairment even if they do not present with overt dysphagia [20,22]. The results of our study are in line with this literature, supporting the idea that screening for swallowing symptoms may offer a low-cost and non-invasive method for identifying individuals at risk of EDS. Furthermore, as the EAT-10 is a self-administered tool that requires minimal clinical training, it has potential utility in workplace health screening and primary care settings.

Lip-seal strength and sleep quality

Reduced lip-seal strength was significantly associated with moderate EDS. Although this may appear counterintuitive at first, it may reflect the nonlinear progression of airway collapse mechanisms and compensatory muscle recruitment. In the earlier or milder stages of upper airway obstruction, a reduction in lip-seal strength may impair oral competence during sleep, contributing to microarousal and fragmented sleep without full apneic episodes. As the condition progresses, more dominant risk factors such as anatomical narrowing, obesity, or neuromuscular decline may overshadow the effects of perioral muscle strength alone [20]. Lip-seal strength is a proxy measure for the strength and coordination of the orbicularis oris and surrounding musculature. These muscles contribute not only to mastication and swallowing, but also to maintaining an airtight oral seal, which is particularly important during nasal breathing [19,23]. Weak lip muscles may increase the likelihood of oral breathing during sleep, which is associated with increased snoring and poor sleep quality [24]. Furthermore, oral breathing can exacerbate airway dryness and inflammation, contributing to a disrupted sleep architecture. Given that lip-seal strength can be trained and improved with specific exercises and devices such as “Ripple-kun,” it represents a modifiable factor that could be targeted in sleep health interventions. Future randomized controlled trials may help clarify whether improving lip-seal strength reduces subjective sleepiness or the frequency of apneic episodes.

Tongue pressure and EDS

Contrary to our expectations, tongue pressure was not significantly associated with moderate or severe EDS. This is in contrast with the findings of several studies that have linked reduced tongue strength to OSA severity, especially in older adults [25]. However, our study population comprised relatively healthy middle-aged working men without OSA, which may have prevented the detection of actual differences. The average age of the subjects was 44.4 years, an age at which age-related changes in muscles involved in tongue pressure, such as the suprahyoid muscles, are not prominently observed. In such populations, the variation in tongue pressure might be insufficient. Further, tongue pressure may not fully capture tongue function during sleep, when muscle tone decreases because of reduced neuromuscular input affected by aging and various diseases [26]. Dynamic assessments, such as polysomnography or endoscopic evaluation during sleep, may be more sensitive to the types of dysfunction relevant to airway patency. It is also possible that the tongue compensates for other oral muscular weaknesses in the early stages of dysfunction, masking its role in the daytime symptoms. Further studies incorporating objective sleep studies and neuromuscular imaging would be helpful to clarify the precise contribution of tongue strength to sleep quality in non-clinical populations.

Snoring as a practical marker

Self-reported snoring was strongly associated with EDS in both severity groups. As a common symptom of OSA and an easily reportable behavior, snoring remains a practical marker of sleep disruption. The strength of its association with ESS scores in our study supports previous literature suggesting that habitual snoring is correlated with sleep fragmentation, oxygen desaturation, and increased sleep latency [27]. While not all individuals who snore exhibit OSA, and not all individuals with OSA snore, the presence of snoring in conjunction with reduced lip-seal strength or difficulty in swallowing should prompt further clinical investigation. Workplace screening programs may benefit from the inclusion of simple snoring questionnaires and oral function tests.

BMI and sleepiness: a complex relationship

BMI was not significantly associated with EDS in our study, which may be inconsistent with the well-established role of obesity in sleep apnea. However, our sample consisted exclusively of male participants younger than 65 years who were otherwise healthy and were likely excluded if they had known sleep disorders. This may have reduced the variability in BMI and the likelihood of detecting its typical effects on sleep apnea risk. In general, BMI is a limited proxy for fat distribution, particularly visceral or pharyngeal fat accumulation, which more directly impacts airway patency [28-30]. Future studies should consider including neck circumference or imaging-based assessments of upper airway anatomy to better explore this relationship.

Implications and future directions

Our findings support the integration of oral functional assessment tools with broader sleep health evaluations in occupational settings. Tools such as the EAT-10 and lip-seal strength measurements are inexpensive, easy to administer, and may help detect early signs of sleep-disordered breathing before the onset of more severe symptoms or comorbidities. Since the ESS used in this study is a screening tool, it is important to receive a definitive diagnosis of OSA. Longitudinal studies are needed to clarify the directionality of these associations, whether oral dysfunction contributes causally to EDS, and whether shared underlying factors affect both. Interventional research can also evaluate the impact of targeted oral muscle training programs on sleep outcomes. Additionally, expanding this research to include female workers, older adults, and individuals with known OSA could help generalize the findings and enable exploration of whether similar patterns exist in other populations.

Limitations

This study had some limitations. First, the cross-sectional nature of this study precludes any inference of causality. Second, we did not perform objective sleep assessments, such as polysomnography, which limited the diagnostic accuracy of true OSA. The questionnaire used was also subjective, so it is possible that it affected the results. Third, our findings may not be generalized beyond healthy working-age Japanese men because women, older adults, and clinical populations were not included. Furthermore, the data obtained may also vary depending on the worker's occupation. Fourth, this study did not investigate oral conditions, such as remaining teeth, xerostomia, or teeth grinding. If groups were formed based on these conditions, the data obtained could differ.

## Conclusions

Swallowing difficulty and reduced lip-seal strength were significantly associated with EDS in working-age Japanese men. Although tongue pressure and BMI were not associated, the findings highlight the importance of oral health in sleep quality. Oral function tests may serve as low-cost, accessible tools for early detection of sleep disorders and for preventive health strategies in workplace settings.

## References

[1] Takano Y, Hirasawa T, Inoue Y (2025). The condition of subjective daytime sleepiness and its related decline in work productivity among daytime workers. J Epidemiol.

[2] Jo JH, Park JW, Jang JH, Chung JW (2022). Hyoid bone position as an indicator of severe obstructive sleep apnea. BMC Pulm Med.

[3] Cui R, Tanigawa T, Sakurai S, Yamagishi K, Iso H (2006). Relationships between sleep-disordered breathing and blood pressure and excessive daytime sleepiness among truck drivers. Hypertens Res.

[4] Kaneita Y, Ohida T (2011). Association of current work and sleep situations with excessive daytime sleepiness and medical incidents among Japanese physicians. J Clin Sleep Med.

[5] Bannai A, Ukawa S, Tamakoshi A (2015). Long working hours and sleep problems among public junior high school teachers in Japan. J Occup Health.

[6] Minoura A, Ihara Y, Kato H (2023). Relationships between lip seal strength, tongue pressure, and daytime sleepiness in Japanese workers: a cross-sectional study. Clin Pract.

[7] Han S, Jee D, Kang YJ, Park YJ, Cho JH (2021). Possible association between oral health and sleep duration: a cross-sectional study based on the Korean National Health and Nutrition Examination Surveys from 2010 to 2015. Medicine (Baltimore).

[8] Zhu KJ, Kuo KT, Heron MJ (2025). Risk factors for obstructive sleep apnea in patients with cleft palate. Ann Plast Surg.

[9] Minoura A, Ihara Y, Kato H (2023). Lip seal strength and tongue pressure among Japanese male workers: comparison of different age groups. Int J Environ Res Public Health.

[10] Johns MW (1991). A new method for measuring daytime sleepiness: the Epworth sleepiness scale. Sleep.

[11] Johns MW (1993). Daytime sleepiness, snoring, and obstructive sleep apnea. The Epworth Sleepiness Scale. Chest.

[12] Takegami M, Suzukamo Y, Wakita T (2009). Development of a Japanese version of the Epworth Sleepiness Scale (JESS) based on item response theory. Sleep Med.

[13] Tang Y, Li D, Yang M (2024). Prevalence of excessive daytime sleepiness (EDS) and its association with quality of life in patients with obstructive sleep apnea (OSA): data from a sleep-center in Shenzhen, a single-center cross-sectional study. J Thorac Dis.

[14] Belafsky PC, Mouadeb DA, Rees CJ, Pryor JC, Postma GN, Allen J, Leonard RJ (2008). Validity and reliability of the Eating Assessment Tool (EAT-10). Ann Otol Rhinol Laryngol.

[15] Ueda T, Oki T, Ohta M, Ogami K, Sakurai K (2019). Intra- and inter investigator reliability of measurement of lip-seal strength in adults. Bull Tokyo Dent Coll.

[16] Oki T, Ohta M, Takano T, Sakurai K, Ueda T (2021). Effective training duration and frequency for lip-seal training in older people using a self-training instrument. Gerodontology.

[17] Siripajana P, Chalidapongse P, Sanguanwong N, Chaweewannakorn C (2024). Efficacy of oropharyngeal exercises as an adjuvant therapy for obstructive sleep apnea: a randomized controlled trial. J Prosthodont Res.

[18] Garbarino S, Durando P, Guglielmi O (2016). Sleep apnea, sleep debt and daytime sleepiness are independently associated with road accidents. A cross-sectional study on truck drivers. PLoS One.

[19] AlShareef SM (2020). Occupational outcomes associated with sleep quality and excessive daytime sleepiness: results from a national survey. Nat Sci Sleep.

[20] Hama Y, Yamada S, Nishimura R (2024). Association between dysphagia risk and sleep quality in community-dwelling older adults: a cross-sectional study. Heliyon.

[21] Schindler A, Mozzanica F, Sonzini G, Plebani D, Urbani E, Pecis M, Montano N (2014). Oropharyngeal dysphagia in patients with obstructive sleep apnea syndrome. Dysphagia.

[22] Campanholo MA, Caparroz FA, Stefanini R, Haddad L, Bittencourt LR, Tufik S, Haddad FL (2021). Dysphagia in patients with moderate and severe obstructive sleep apnea. Braz J Otorhinolaryngol.

[23] Zhao M, Mohamed AS, Cheng B (2025). A nomogram for assisting in diagnosing mouth breathing based on maxillofacial surface electromyographic activity. BMC Oral Health.

[24] Miyakawa T, Iwasaki T, Suga H, Ban Y, Yamasaki Y (2018). Survey regarding effects of mouth breathing in children on sleep and daytime activities. Jpn J Pediatric Dentistry.

[25] Lee J, Makihara E, Watanabe T (2024). Sleep status and tongue pressure in patients with obstructive sleep apnea. J Oral Sleep Med.

[26] McSharry DG, Saboisky JP, Deyoung P (2014). Physiological mechanisms of upper airway hypotonia during REM sleep. Sleep.

[27] Hoffstein V (1995). Snoring and nocturnal oxygenation. Is there a relationship?. Chest.

[28] Mortimore IL, Marshall I, Wraith PK, Sellar RJ, Douglas NJ (1998). Neck and total body fat deposition in nonobese and obese patients with sleep apnea compared with that in control subjects. Am J Respir Crit Care Med.

[29] Shinohara E, Kihara S, Yamashita S (1997). Visceral fat accumulation as an important risk factor for obstructive sleep apnoea syndrome in obese subjects. J Intern Med.

[30] D'Angelo GF, de Mello AA, Schorr F (2023). Muscle and visceral fat infiltration: a potential mechanism to explain the worsening of obstructive sleep apnea with age. Sleep Med.

